# Supplementary material for: Effects of Multidisciplinary Biopsychosocial Rehabilitation on Short-Term Pain and Disability in Chronic Low Back Pain: A Systematic Review with Network Meta-Analysis
Source: J Clin Med. 2023 Dec 4;12(23):7489. doi: 10.3390/jcm12237489 (PMC10707228; doi:10.3390/jcm12237489)

Supplement document S1. Search strategy

PubMed – articles retrieved up to 4th of March, 2022.

#	**Search term**	**Search Details as generated by Pubmed**	**Items**
1	low back pain	"low back pain"[MeSH Terms] OR ("low"[All Fields] AND "back"[All Fields] AND "pain"[All Fields]) OR "low back pain"[All Fields]	12565
2	exercise therapy	"exercise therapy"[MeSH Terms] OR ("exercise"[All Fields] AND "therapy"[All Fields]) OR "exercise therapy"[All Fields]	88380
3	cognitive behavior therapy [MeSH Terms]	"cognitive behavioral therapy"[MeSH Terms]	14463
4	multidisciplinary biopsychosocial rehabilitation	("interdisciplinary studies"[MeSH Terms] OR ("interdisciplinary"[All Fields] AND "studies"[All Fields]) OR "interdisciplinary studies"[All Fields] OR "multidisciplinary"[All Fields]) AND ("biopsychosocial"[All Fields] OR "biopsychosocially"[All Fields]) AND ("rehabilitant"[All Fields] OR "rehabilitants"[All Fields] OR "rehabilitate"[All Fields] OR "rehabilitated"[All Fields] OR "rehabilitates"[All Fields] OR "rehabilitating"[All Fields] OR "rehabilitation"[MeSH Terms] OR "rehabilitation"[All Fields] OR "rehabilitations"[All Fields] OR "rehabilitative"[All Fields] OR "rehabilitation"[MeSH Subheading] OR "rehabilitation s"[All Fields] OR "rehabilitational"[All Fields] OR "rehabilitator"[All Fields] OR "rehabilitators"[All Fields])	913
5	Psychosocial Support Systems [MeSH Terms]	"psychosocial support systems"[MeSH Terms]	278
6	Occupational Therapy[MeSH Terms]	"occupational therapy"[MeSH Terms]	34439
7	Patient Education[MeSH Terms]	"patient education as topic"[MeSH Terms]	143191
8	Biofeedback[MeSH Terms]	"biofeedback, psychology"[MeSH Terms])	355
9	#2 OR #3 OR #4 OR #5 OR #6 OR #7 OR #8	/	48706
10	#1 OR #9	/	**1210***

*** Filters: Controlled Clinical Trial, Pragmatic Clinical Trial, Randomized Controlled Trial**

Ovid – articles retrieved up to 4th of March 2022.
(Journals@Ovid Full Text & Cochrane Central Register of Controlled Trials, CINAHAL, PsycINFO)

#	**Search term**	**No. of papers**
1	randomized controlled trial.pt.	545949
2	controlled clinical trial.pt.	92954
3	random*.ti,ab.	1516760
4	or/1-3	1725090
5	exp back pain/ or exp low back pain/	5465
6	chronic low back pain.mp.	15053
7	dorsalgia.mp.	224
8	lumbago.mp.	1544
9	or/5-8	20228
10	exp exercise therapy/ or exp motion therapy, continuous passive/ or exp muscle stretching exercises/ or exp plyometric exercise/ or exp resistance training/	15499
11	exercise thera$.mp.	22105
12	10 or 11	26266
13	Multidisciplinary Biopsychosocial Rehabilitation.mp. [mp=ti, ab, tx, ct, ot, sh, hw, kw]	107
14	exp social support/	3466
15	exp occupational therapy/	813
16	exp Patient Education/	9173
17	exp Behavior Therapy/	10077
18	exp biofeedback, psychology/	1494
19	or/13-18	22453
20	4 and 9	8402
21	12 or 19	47513
22	20 and 21	**1354**

Ovid – articles retrieved up to 4th of March 2022.
(Journals@Ovid Full Text & Cochrane Central Register of Controlled Trials, EMBASE)

#	**Search term**	**No. of papers**
1	randomized controlled trial.pt.	0
2	controlled clinical trial.pt.	0
3	random*.ti,ab.	1482762
4	or/1-3	1482762
5	exp back pain/ or exp low back pain/	6530
6	chronic low back pain.mp.	15050
7	dorsalgia.mp.	237
8	lumbago.mp.	1601
9	or/5-8	20984
10	exp exercise therapy/ or exp motion therapy, continuous passive/ or exp muscle stretching exercises/ or exp plyometric exercise/ or exp resistance training/	17648
11	exercise thera$.mp.	23263
12	10 or 11	28475
13	Multidisciplinary Biopsychosocial Rehabilitation.mp. [mp=ti, ab, tx, ct, ot, sh, hw, kw]	107
14	exp social support/	4142
15	exp occupational therapy/	947
16	exp Patient Education/	9962
17	exp Behavior Therapy/	23040
18	exp biofeedback, psychology/	1808
19	or/13-18	5188
20	4 and 9	7840
21	12 or 19	33447
22	20 and 21	**1860**

PEDro

Therapy: fitness training
Problem: pain
Body part: Lumbar spine, sacroiliac joint or pelvis
Topic: chronic pain
Method: clinical trial

Match all search terms (AND)

= 195 (04.03.2022.)

Supplement document S2. Risk of Bias 2 Overall category explained

The overall risk of bias in our network meta-analysis was determined by an algorithm that considered the importance and level of bias across different domains, namely D1, D3, and D4. The risk of bias was classified into three categories: Low Bias (LB), Some Concerns (SC), and High Bias (HB). Domains D1, D3, and D4 were classified as most having the most impact on risk of bias in eligible studies. In the methodology of our analysis, we identified D1, D3, and D4 as the most impactful domains for the bias assessment due to their inherent attributes in study design and data management. We recognized D2 (Bias due to deviations from intended interventions) and D5 (Bias in selection of the reported result) as important but slightly less influential in determining the overall bias for several reasons. For D2, it's critical to acknowledge the complex nature of many interventions in medical research, especially those that span a long duration. In these contexts, minor deviations from the intended intervention protocol are somewhat inevitable. Further, blinding of caregivers is often impracticable due to the nature of the interventions. These factors decrease the relative weight of D2 in bias determination as deviations may occur without significantly compromising the reliability of the study results.

For D5, the assessment of bias in the selection of reported results can be challenging due to the lack of publicly available pre-study protocols, particularly for older studies. This limitation often restricts our ability to definitively determine if the re-ported outcomes were selected based on the results.

Despite the differential weighting of these domains, it is essential to stress that all were considered in our overall risk of bias assessment, however D1, D3, and D4 generally tend to carry more weight due to their more direct influence on study outcomes and results.

1. The studies were classified as having "Low Bias" if all domains were assessed as LB or if domains D2 and/or D5 were assessed as having some concerns, but all other domains were LB.
2. The classification "Some Concerns" was assigned if either D1, D3, or D4 were assessed as having some concerns or if all other domains were LB while D2 and/or D5 were rated as high bias.
3. A classification of "High Bias" was assigned under the following conditions: if either D1, D3, or D4 were rated as high bias, if D1, D3, or D4 were assessed as having some concerns and D2 and D5 were high bias, or under any other combination not fitting the previous categories.

Figure S1. PRISMA flow diagram

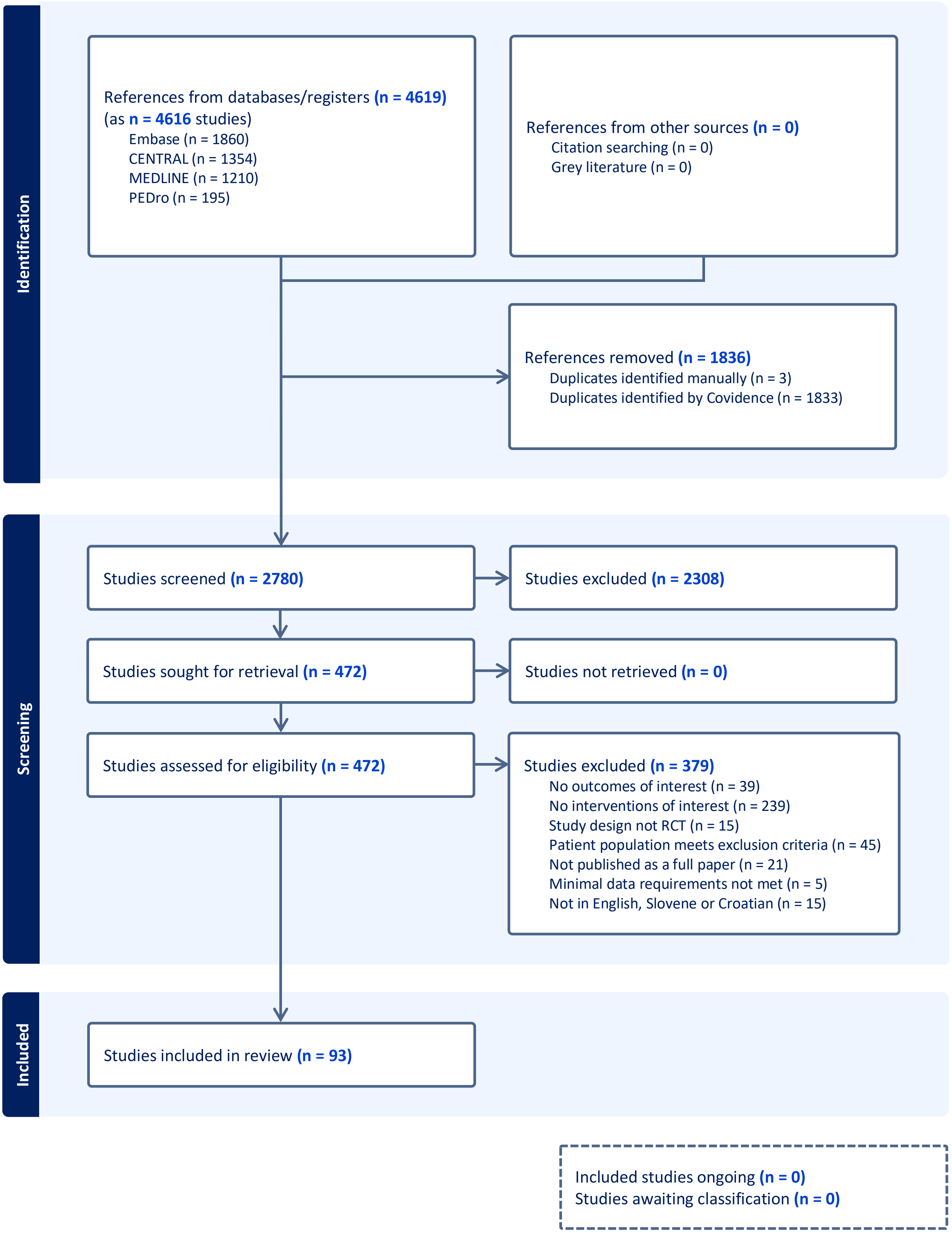

22nd July 2023

covidence

Table S1. Characteristics of included studies (k = 93)

Author, year	Pain outcomes	Disability outcomes	Comparison	# of participants	Duration of follow-up	Setting
Ahmed 2021	VAS (0-10)	ODI	ET vs MBR vs UC	125	12 weeks	Clinical inpatient
Almhdawi 2020	VAS (0-10)	ODI	ET vs MI	41	6 weeks	Outpatient
Alvani 2021	VAS (0-10)	ODI	ET vs MI	30	8 weeks	Clinical inpatient
Barone Gibbs 2018	VAS (0-10)	ODI	MBR vs MI	27	4 weeks	Outpatient
Bodes Pardo 2018	VAS (0-10)	RMDQ	ET vs MBR	56	4 weeks	Clinical inpatient
Borys 2015	VAS (0-10)	//	MBR vs MI	132	3 weeks	Clinical inpatient
Costa 2009	VAS (0-10)	RMDQ	ET vs UC	154	8 weeks	Clinical inpatient
Costantino 2014	//	RMDQ	ET vs MBR	54	12 weeks	Clinical inpatient
Cruz-Diaz 2018	VAS (0-10)	RMDQ	ET vs MI	61	16 weeks	University laboratory
Cuesta-Vargas 2012	VAS (0-100)	RMDQ	ET vs UC	58	15 weeks	Clinical inpatient
Darnall 2021	VAS (0-10)	//	MBR vs MI	218	2 weeks	Academic
Devasahayam 2014	VAS (0-10)	RMDQ	MBR vs UC	15	4 weeks	Clinical inpatient
Donzelli 2006	VAS (0-10)	ODI	ET vs MBR	43	4 weeks	Outpatient
Dufour 2010	VAS (0-100)	RMDQ	ET vs MBR	272	12 weeks	Clinical inpatient
Durmus 2014	VAS (0-10)	ODI	ET vs MBR	121	12 weeks	Clinical inpatient
Frost 1995	VAS (0-100)	ODI	ET vs MI	71	4 weeks	Clinical inpatient
Garcia 2018	VAS (0-10)	RMDQ	MI vs MBR	147	5 weeks	Clinical inpatient
Gardner 2019	VAS (0-10)	QBPDS	ET vs MBR	75	8 weeks	Clinical inpatient
Godfrey 2020	VAS (0-10)	RMDQ	MBR vs UC	217	12 weeks	Clinical inpatient
Goldby 2006	VAS (0-100)	ODI	MI vs MBR	124	10 weeks	Clinical inpatient
Hall 2011	VAS (0-10)	RMDQ	ET vs MI	160	10 weeks	Clinical inpatient
Haufe 2017	VAS (0-10)	ODI	ET vs vs MI	55	20 weeks	Clinical inpatient
Highland 2018	VAS (0-10)	RMDQ	ET vs vs MI	68	8 weeks	Military hospital
Jaromi 2012	VAS (0-100)	//	UC vs MBR	111	6 weeks	University clinic
Jaromi 2018	VAS (0-100)	//	MBR vs MI	137	12 weeks	Academic
Jensen 2012	VAS (0-10)	RMDQ	ET vs MI	100	10 weeks	Clinical inpatient
Kääpä 2006	VAS (0-10)	ODI	ET vs MBR	120	8 weeks	Clinical inpatient

Author, year	Pain outcomes	Disability outcomes	Comparison	# of participants	Duration of follow-up	Setting
Kader 2012	MGPQ (0-78)	ODI	ET vs MBR	42	10 weeks	Clinical inpatient
Kankaanpää 1999	VAS (0-100)	PDI	ET vs MI	54	12 weeks	Clinical inpatient
Khan 2014	VAS (0-10)	RMDQ	UC vs MBR	54	12 weeks	Clinical inpatient
Khodadad 2020	VAS (0-10)	//	ET vs UC vs MBR	52	8 weeks	Clinical inpatient
Kim 2020	VAS (0-10)	ODI	ET vs MBR	48	8 weeks	Clinical inpatient
Kim 2022	VAS (0-10)	RMDQ	ET vs MBR	35	8 weeks	Clinical inpatient
Kofotolis 2006	VAS (0-10)	ODI	ET vs UC	86	4 weeks	Clinical inpatient
Koldas Dogan 2008	VAS (0-100)	RMDQ	ET vs UC	55	6 weeks	Outpatient
Kumar 2009 A	VAS (0-10)	//	ET vs UC	102	4 weeks	Clinical inpatient
Kumar 2009 B	VAS (0-10)	//	ET vs UC	30	4 weeks	Athletic club
Kuvacic 2018	VAS (0-10)	ODI	ET vs MI	30	8 weeks	Clinical inpatient
Linden 2014	VAS (0-10)	//	MBR vs UC	103	3 weeks	Clinical inpatient
Masharawi 2013	VAS (0-10)	RMDQ	ET vs MI	40	4 weeks	Clinical inpatient
Mazloum 2018	VAS (0-10)	ODI	ET vs MI	56	4 weeks	Clinical inpatient
McCaskey 2018	VAS (0-100)	ODI	ET vs UC	22	5 weeks	Clinical inpatient
Michalsen 2021	VAS (0-100)	RMDQ	ET vs UC	182	8 weeks	Clinical inpatient
Miyamoto 2013	VAS (0-10)	RMDQ	ET vs MI	86	6 weeks	Clinical inpatient
Monticone 2013	VAS (0-10)	//	ET vs MBR	90	5 weeks	Clinical inpatient
Monticone 2014	VAS (0-10)	ODI	UC vs MBR	20	8 weeks	Clinical inpatient
Monticone 2016	VAS (0-10)	ODI	UC vs MBR	150	5 weeks	Outpatient
Morone 2011	VAS (0-10)	ODI	MI vs MBR	70	4 weeks	Clinical inpatient
Morone 2012	VAS (0-10)	ODI	MBR vs MI	50	4 weeks	Clinical inpatient
Moseley 2002	VAS (0-10)	RMDQ	MI vs MBR	57	4 weeks	University clinic
Nambi 2021	VAS (0-10)	//	ET vs UC	54	4 weeks	Clinical inpatient
Narouei 2020	VAS (0-10)	ODI	ET vs UC	34	4 weeks	Clinical inpatient
Nassif 2011	VAS (0-10)	RMDQ	MI vs MBR	75	8 weeks	Outpatient
Natour 2015	VAS (0-10)	RMDQ	ET vs MI	60	7 weeks	Clinical inpatient
Nicholas 1991	PRC (0-5)	//	MBR vs UC	59	5 weeks	Clinical inpatient
Okafor 2012	VAS (0-10)	//	ET vs UC	30	6 weeks	Clinical inpatient

Author, year	Pain outcomes	Disability outcomes	Comparison	# of participants	Duration of follow-up	Setting
O'Keeffe 2020	VAS (0-10)	ODI	MBR vs UC	194	8 weeks	Clinical inpatient
Patti 2016	//	ODI	ET vs MI	38	14 weeks	Clinical inpatient
Paungmali 2017	VAS (0-10)	//	ET vs MI	50	1 week	Controlled laboratory
Petrozzi 2019	VAS (0-10)	RMDQ	UC vs MBR	108	6 weeks	Outpatient
Phattharasupharerk 2019	VAS (0-100)	RMDQ	ET vs MI	72	6 weeks	Outpatient
Pires 2015	VAS (0-100)	QBPDS	ET vs MBR	62	6 weeks	Clinical inpatient
Polaski 2021	VAS (0-10)	RMDQ	MBR vs MI	38	4 weeks	Clinical inpatient
Rabiei 2021	VAS (0-10)	RMDQ	ET vs MBR	73	8 weeks	Clinical inpatient
Roche-Leboucher 2011	VAS (0-10)	//	ET vs MBR	132	5 weeks	Clinical inpatient
Rydeard 2006	VAS (0-100)	RMDQ	ET vs Uc	39	4 weeks	Clinical inpatient
De Sousa 2009	VAS (0-10)	RMDQ	MBR vs MI	52	8 weeks	Outpatient
Santos 2022	VAS (0-10)	RMDQ	ET vs MI	43	4 weeks	Clinical inpatient
Saper 2009	VAS (0-10)	RMDQ	ET vs MI	30	12 weeks	Clinical inpatient
Saper 2017	VAS (0-10)	RMDQ	ET vs MI	320	12 weeks	Clinical inpatient
Schinhan 2016	VAS (0-10)	//	ET vs MI	30	8 weeks	Clinical inpatient
Shamsi 2016	VAS (0-100)	ODI	ET vs UC	43	5 weeks	Clinical inpatient
Shaughnessy 2004	//	RMDQ	ET vs MI	41	10 weeks	Clinical inpatient
Sherman 2011	VAS (0-10)	RMDQ	ET vs MI	228	12 weeks	Clinical inpatient
Tilbrook 2011	//	RMDQ	ET vs UC	299	12 weeks	Clinical inpatient
Torstensen 1998	VAS (0-100)	ODI	ET vs UC	138	12 weeks	Clinical inpatient
Tritilanunt 2001	VAS (0-10)	//	ET vs MBR	68	12 weeks	Outpatient
Turner 1990	MGPQ (0-78)	//	ET vs MBR vs MI	96	8 weeks	Clinical inpatient
Valenza 2017	VAS (0-10)	RMDQ	ET vs MI	54	8 weeks	Clinical inpatient
Van Der Roer 2008	VAS (0-10)	RMDQ	UC vs MBR	114	6 weeks	Clinical inpatient
Van Erp 2021	VAS (0-10)	QBPDS	UC vs MBR	25	12 sessions	Clinical inpatient
Vibe Fersum 2013	VAS (0-10)	ODI	MBR vs UC	94	12 weeks	Outpatient
Vollenbroek-Hutten 2004	//	RMDQ	MI vs MBR	151	8 weeks	Outpatient
Walti 2015	VAS (0-10)	RMDQ	UC vs MBR	28	8 weeks	Clinical inpatient
Waseem 2019	//	ODI	ET vs UC	108	6 weeks	Clinical inpatient

Author, year	Pain outcomes	Disability outcomes	Comparison	# of participants	Duration of follow-up	Setting
Weifen 2013	VAS (0-100)	//	ET vs MI	188	12 weeks	Clinical inpatient
Williams 2009	VAS (0-100)	ODI	ET vs MI	90	12 weeks	Clinical inpatient
Williams 2018	VAS (0-10)	RMDQ	MBR vs MI	159	12 weeks	Outpatient
Yang 2021	VAS (0-100)	RMDQ	ET vs UC	39	8 weeks	Clinical inpatient
Zadro 2019	VAS (0-10)	RMDQ	ET vs UC	60	8 weeks	Clinical inpatient
Zhang 2014	VAS (0-10)	ODI	UC vs MBR	54	12 weeks	Clinical inpatient
Zheng 2022	VAS (0-10)	RMDQ	ET vs MBR	40	6 weeks	Clinical inpatient
Zou 2019	VAS (0-10)	//	ET vs MI	43	12 weeks	Clinical inpatient

Legend: VAS = visual analogue scale; PRC - Pain Rating Chart; ODI = Oswestry Disability Index; RMDQ = Roland – Morris Disability Questionnaire; QBPDS = Quebec Back Pain Disability Scale; PDI = Pain and Disability Index; DI = Disability Index; HFAQ - Hannover Functional Ability Questionnaire; MGPQ = McGill Pain Questionnaire

Figure S2. Risk of Bias 2 visualization (part 1)

	Risk of bias domains					
	D1	D2	D3	D4	D5	Overall
Ahmed 2021	+	-	+	+	-	-
Almhdawi 2020	-	X	+	X	+	X
Alvani 2021	+	-	+	X	X	X
Barone Gibbs 2018	X	X	X	X	-	X
Bodes Pardo 2018	+	-	+	+	-	+
Borys 2015	X	-	+	+	+	X
Costa 2009	+	-	+	+	+	+
Costantino 2014	-	X	+	-	+	X
Cruz-Diaz 2018	+	+	+	-	+	-
Cuesta-Vargas 2012	+	X	+	-	-	X
Darnall 2021	+	-	+	+	+	+
Devasahayam 2014	+	-	+	+	+	+
Donzelli 2006	X	X	X	X	X	X
Dufour 2010	+	-	+	+	+	+
Durmus 2014	+	-	+	+	-	-
Frost 1995	+	-	+	+	+	+
Garcia 2018	+	-	+	+	+	+
Gardner 2019	-	-	+	+	+	-
Godfrey 2020	+	+	+	+	-	+
Goldby 2006	+	X	X	-	+	X

Figure S3. Risk of Bias 2 visualization (part 2)

Hall 2011	+	X	+	-	+	X
Haufe 2017	-	X	+	X	-	X
Highland 2018	-	+	+	-	+	-
Jaromi 2012	-	X	+	-	+	X
Jaromi 2018	X	-	+	-	+	X
Jensen 2012	+	-	+	+	+	+
Kääpä 2006	+	X	+	-	+	X
Kader 2012	-	X	+	-	+	X
Kankaanpää 1999	+	X	+	-	+	X
Khan 2014	-	X	+	-	-	X
Khodadad 2020	+	-	+	+	+	+
Kim 2020	X	X	X	X	-	X
Kim 2022	-	-	+	X	+	X
Kofotolis 2006	X	X	-	-	+	X
Koldas Dogan 2008	X	X	-	-	+	X
Kumar 2009 A	-	X	+	-	+	X
Kumar 2009 B	-	X	+	-	+	X
Kuvacic 2018	+	-	+	+	+	+
Linden 2014	-	-	+	+	+	-
Masharawi 2013	+	X	+	-	+	X
Mazloum 2018	X	+	+	-	+	X
McCaskey 2018	+	-	+	+	+	+
Michalsen 2021	+	+	+	-	+	-
Miyamoto 2013	+	-	+	+	+	+
Monticone 2013	+	-	+	X	X	X

Figure S4. Risk of Bias 2 visualization (part 3)

Morone 2012	+	-	+	+	+	+
Moseley 2002	X	X	X	X	-	X
Nambi 2021	+	-	+	+	+	+
Narouei 2020	+	-	+	+	-	-
Nassif 2011	+	-	X	X	-	X
Natour 2015	+	X	+	-	+	X
Nicholas 1991	+	-	X	X	X	X
Okafor 2012	-	X	-	-	+	X
O'Keeffe 2020	+	-	+	X	-	X
Patti 2016	+	+	+	+	-	+
Paungmali 2017	X	+	+	-	+	X
Petrozzi 2019	+	+	+	-	+	-
Phattharasupharerk 2019	+	+	+	-	+	-
Pires 2015	+	+	+	X	+	X
Polaski 2021	+	-	-	+	+	-
Rabiei 2021	+	X	-	X	X	X
Roche-Leboucher 2011	-	X	-	-	+	X
Rydeard 2006	+	-	+	+	+	+
De Sousa 2009	X	X	+	+	+	X
Santos 2022	+	-	+	-	+	+
Saper 2009	+	-	X	X	-	X
Saper 2017	X	X	+	+	+	X
Schinhan 2016	+	+	+	+	-	+
Shamsi 2016	X	-	+	-	+	X

Figure S5. Risk of Bias 2 visualization (part 4)

Shaughnessy 2004	X	X	+	-	+	X
Sherman 2011	+	-	+	+	+	+
Tilbrook 2011	+	X	+	-	+	X
Torstensen 1998	+	X	+	+	+	-
Tritilanunt 2001	-	X	+	-	-	X
Turner 1990	+	X	X	-	+	+
Valenza 2017	+	+	+	-	+	-
VanDerRoer 2008	+	-	+	-	-	-
vanErp 2021	+	-	X	X	-	X
VibeFersum 2013	+	X	+	+	+	-
Vollenbroek-Hutten 2004	-	X	+	+	+	X
Walti 2015	+	+	+	+	-	+
Waseem 2019	-	X	X	X	X	X
Weifen 2013	-	X	+	+	+	X
Williams 2009	+	X	+	+	+	-
Williams 2018	+	+	+	X	+	X
Yang 2021	-	-	+	X	-	X
Zadro 2019	+	-	+	+	+	+
Zhang 2014	X	X	X	X	X	X
Zheng 2022	+	+	+	+	+	+
Zou 2019	+	-	+	+	+	+

Domains:
D1: Bias arising from the randomization process.
D2: Bias due to deviations from intended intervention.
D3: Bias due to missing outcome data.
D4: Bias in measurement of the outcome.
D5: Bias in selection of the reported result.

Judgement
X High
- Some concerns
+ Low

Table S2. Mean difference and standard error of pain outcomes

ID	MD	SE (MD)	Treatment 1	Treatment 2
Ahmed 2021[1]	-1.6	2.57	ET	MBR-WR
Ahmed 2021[1]	-1.8	2.68	ET	UC
Ahmed 2021[1]	-0.2	2.52	MBR-WR	UC
Almhdawi 2020	-27.0	6.40	ET	MI
Alvani 2021	-14.6	6.06	ET	MI
Barone Gibbs 2018	4.0	8.30	MBR-ED	MI
Bodes Pardo 2018	18.0	4.33	ET	MBR-ED
Borys 2015	-15.0	2.82	MBR-ED	MI
Costa 2009	-10.0	4.35	ET	UC
Cruz-Diaz 2018	-28.0	3.22	ET	MI
Cuesta-Vargas 2012	-14.9	4.37	ET	UC
Darnall 2021	-10.7	3.60	MBR-BE	MI
Devasahayam 2014	10.0	12.23	MBR-BE	UC
Donzelli 2006	4.4	5.23	ET	MBR-ED
Dufour 2010	6.2	2.52	ET	MBR-ED
Durmus 2014	23.7	3.31	ET	MBR-ED
Frost 1995	-10.0	3.78	ET	MI
Garcia 2018	-8.6	4.58	MBR-ED	MI
Gardner 2019	23.0	4.16	ET	MBR-BE
Godfrey 2020	-5.0	3.39	MBR-BE	UC
Goldby 2006	-5.6	6.78	MBR-ED	MI
Hall 2011	-13.0	3.38	ET	MI
Haufe 2017	-16.0	6.20	ET	MI
Highland 2018	-11.9	4.94	ET	MI
Jaromi 2012	-1.3	0.80	MBR-ED	UC
Jaromi 2018	-41.8	1.37	MBR-ED	MI
Jensen 2012	-5.0	4.30	ET	MI
Kääpä 2006	1.0	4.48	ET	MBR-BE
Kader 2012	13.8	5.35	ET	MBR-ED
Kankaanpää 1999	-8.3	7.01	ET	MI
Khan 2014	-25.9	3.65	MBR-ED	UC
Khodadad 2020[2]	1.0	3.86	ET	MBR-BE
Khodadad 2020[2]	-25.0	5.36	MBR-BE	UC
Khodadad 2020[2]	-24.0	5.00	ET	UC
Kim 2020	3.7	4.64	ET	MBR-ED
Kim 2022	5.9	4.46	ET	MBR-ED
Kofotolis 2006	-2.0	1.12	ET	UC
Koldas Dogan 2008	-3.2	8.13	ET	UC
Kumar 2009 A	-12.2	1.54	ET	UC
Kumar 2009 B	-8.7	3.32	ET	UC
Kuvacic 2018	-12.0	2.15	ET	MI
Linden 2014	-10.4	3.81	MBR-BE	UC
Masharawi 2013	-22.0	3.90	ET	MI
Mazloum 2018	-22.5	4.06	ET	MI
McCaskey 2018	0.0	7.08	ET	UC
Michalsen 2021	-3.3	2.21	ET	UC

ID	MD	SE (MD)	Treatment 1	Treatment 2
Miyamoto 2013	-21.0	4.96	ET	UC
Monticone 2013	22.7	2.38	ET	MBR-BE
Monticone 2014	0.0	7.07	MBR-BE	UC
Monticone 2016	-31.0	2.51	MBR-BE	UC
Morone 2011	-18.0	4.66	MBR-ED	MI
Morone 2012	-20.0	6.11	MBR-WR	MI
Moseley 2002	-12.3	5.35	MBR-ED	MI
Nambi 2021	-11.0	1.44	ET	UC
Narouei 2020	-11.0	5.35	ET	UC
Nassif 2011	-16.5	6.32	MBR-ED	MI
Natour 2015	-4.6	7.04	ET	UC
Nicholas 1991	-12.9	4.34	MBR-BE	UC
Okafor 2012	-15.7	4.40	ET	UC
O'Keeffe 2020	-16.9	4.28	MBR-BE	UC
Paungmali 2017	-5.5	5.94	ET	MI
Petrozzi 2019	-1.0	3.89	MBR-BE	UC
Phattharasupharerk 2019	-39.5	4.88	ET	MI
Pires 2015	6.1	4.84	ET	MBR-ED
Polaski 2021	-8.5	5.33	MBR-WR	MI
Rabiei 2021	11.2	3.25	ET	MBR-ED
Roche-Leboucher 2011	5.0	3.99	ET	MBR-WR
Rydeard 2006	-15.6	4.74	ET	UC
De Sousa 2009	-14.1	7.36	MBR-BE	MI
Santos 2022	-14.6	5.07	ET	MI
Saper 2009	-27.0	5.94	ET	MI
Saper 2017	-4.9	3.06	ET	MI
Schinhan 2016	-16.4	5.68	ET	UC
Shamsi 2016	0.0	3.92	ET	UC
Sherman 2011	-1.0	3.76	ET	MI
Torstensen 1998	-1.8	4.55	ET	UC
Tritilanunt 2001	16.7	4.51	ET	MBR-ED
Turner 1990[3]	1.6	4.56	ET	MBR-BE
Turner 1990[3]	-6.0	4.73	MBR-BE	MI
Turner 1990[3]	-4.4	4.23	ET	MI
Valenza 2017	-14.0	5.17	ET	UC
Van Der Roer 2008	-1.0	4.53	MBR-BE	UC
Van Erp 2021	-2.0	10.66	MBR-BE	UC
VibeFersum 2013	-21.0	3.75	MBR-BE	UC
Walti 2015	-12.3	6.63	MBR-ED	UC
Weifen 2013	-9.0	0.71	ET	MI
Williams 2009	-8.5	4.64	ET	MI
Williams 2018	0.0	3.60	MBR-ED	MI
Yang 2021	-1.1	6.99	ET	UC
Zadro 2019	-6.0	6.07	ET	UC
Zhang 2014	-6.9	4.99	MBR-ED	UC
Zheng 2022	-1.0	4.30	ET	MBR-ED
Zou 2019	-19.8	3.38	ET	MI

Table S3. Standardized Mean difference and standard error of disability outcomes

ID	SMD	SE (SMD)	Treatment 1	Treatment 2
Ahmed 2021[1]	0.054	0.224	ET	MBR-WR
Ahmed 2021[1]	0.167	0.219	ET	UC
Ahmed 2021[1]	0.113	0.216	MBR-WR	UC
Almhdawi 2020	-0.836	0.327	ET	MI
Alvani 2021	-0.684	0.377	ET	MI
Barone Gibbs 2018	-0.347	0.389	MBR-ED	MI
Bodes Pardo 2018	1.282	0.295	ET	MBR-ED
Costa 2009	-0.369	0.163	ET	UC
Costantino 2014	-0.453	0.276	ET	MBR-ED
Cruz-Diaz 2018	-2.159	0.323	ET	MI
Cuesta-Vargas 2012	-0.783	0.297	ET	UC
Devasahayam 2014	-2.035	0.679	MBR-BE	UC
Donzelli 2006	0.163	0.306	ET	MBR-ED
Dufour 2010	0.255	0.128	ET	MBR-ED
Durmus 2014	0.917	0.191	ET	MBR-ED
Frost 1995	-0.330	0.239	ET	MI
Garcia 2018	-0.295	0.166	MBR-ED	MI
Gardner 2019	0.700	0.238	ET	MBR-BE
Godfrey 2020	-0.218	0.143	MBR-BE	UC
Goldby 2006	0.168	0.200	MBR-ED	MI
Gupta 2019	-7.023	1.036	MBR-WR	UC
Hall 2011	-0.209	0.159	ET	MI
Haufe 2017	-0.429	0.273	ET	MI
Highland 2018	-0.489	0.246	ET	MI
Jensen 2012	0.019	0.215	ET	MI
Kääpä 2006	0.065	0.183	ET	MBR-BE
Kader 2012	0.048	0.309	ET	MBR-ED
Kankaanpää 1999	-0.009	0.274	ET	MI
Khan 2014	-1.956	0.335	MBR-ED	UC
Kim 2020	0.347	0.362	ET	MBR-ED
Kim 2022	0.582	0.341	ET	MBR-ED
Kofotolis 2006	-0.914	0.277	ET	UC
Koldas Dogan 2008	-0.729	0.345	ET	UC
Kuvacic 2018	-0.995	0.390	ET	MI
Masharawi 2013	-0.857	0.332	ET	MI
Mazloum 2018	-0.663	0.339	ET	MI
McCaskey 2018	-0.407	0.432	ET	UC
Michalsen 2021	0.110	0.171	ET	UC
Miyamoto 2013	-0.739	0.223	ET	MI
Monticone 2013	2.756	0.297	ET	MBR-BE
Monticone 2014	-1.858	0.553	MBR-BE	UC
Monticone 2016	-1.889	0.198	MBR-BE	UC
Morone 2011	-0.014	0.243	MBR-ED	UC
Morone 2012	-0.376	0.286	MBR-WR	MI
Moseley 2002	-0.633	0.293	MBR-ED	MI
Narouei 2020	-0.826	0.371	ET	UC

ID	SMD	SE (SMD)	Treatment 1	Treatment 2
Nassif 2011	-0.201	0.259	MBR-ED	MI
Natour 2015	-0.373	0.261	ET	UC
O'Keeffe 2020	-0.825	0.183	MBR-BE	UC
Patti 2016	-0.296	0.326	ET	MI
Petrozzi 2019	-0.088	0.194	MBR-BE	UC
Phattharasupharerk 2019	-0.581	0.241	ET	MI
Pires 2015	0.460	0.271	ET	MBR-ED
Polaski 2021	-0.746	0.337	MBR-WR	MI
Rabiei 2021	0.560	0.239	ET	MBR-ED
Rydeard 2006	-0.768	0.334	ET	UC
De Sousa 2009	-0.509	0.282	MBR-BE	UC
Santos 2022	-3.678	0.649	ET	MI
Saper 2009	-0.924	0.387	ET	MI
Saper 2017	-0.215	0.157	ET	MI
Shamsi 2016	-0.440	0.309	ET	UC
Shaughnessy 2004	-1.363	0.350	ET	MI
Sherman 2011	-0.252	0.188	ET	MI
Tilbrook 2011	-0.399	0.121	ET	UC
Torstensen 1998	-0.053	0.170	ET	UC
Valenza 2017	-0.826	0.284	ET	MI
Van Der Roer 2008	0.000	0.208	MBR-BE	UC
VanErp 2021	-0.346	0.404	MBR-BE	UC
VibeFersum 2013	-1.466	0.234	MBR-BE	UC
Vollenbroek-Hutten 2004	-0.398	0.165	MBR-ED	MI
Walti 2015	-0.695	0.398	MBR-ED	UC
Waseem 2019	-1.277	0.212	ET	UC
Williams 2009	-0.284	0.228	ET	MI
Williams 2018	-0.298	0.180	MBR-ED	MI
Yang 2021	0.063	0.320	ET	UC
Zadro 2019	-0.333	0.260	ET	UC
Zhang 2014	-0.420	0.289	MBR-ED	UC
Zheng 2022	-0.259	0.318	ET	MBR-ED

Legend (S2 & S3): MD = mean difference; SMD = standardized mean difference; SE (MD) = standard error of mean difference; SE (SMD) = standard error of standardized mean difference; [1] = three-arm study Ahmed, 2021; [2] = three-arm study Khodadad 2020; [3] = three-arm study Turner 1990

Note 1: Difference is calculated as Treatment 1 – Treatment 2
Note 2: Mean difference is on a scale of 0 – 100 (VAS pain)

Frequentist Network meta-analysis of pain outcomes

Table S4. Between. within and total homogeneity / consistency

	Q	Df	p-value
Total	920.85	85	<0.001
Within designs	628.07	74	<0.001
Between designs	292.78	11	<0.001

Table S5. Q statistic under the assumption of a full design-by-treatment interaction random effects model

	**Q**	**Df**	**p-value**	**τ**	**τ2**
Between designs	14.39	11	0.212	9.359	87.590

Figure S6. Local inconsistency assessment using inconsistency factors and 95% CI

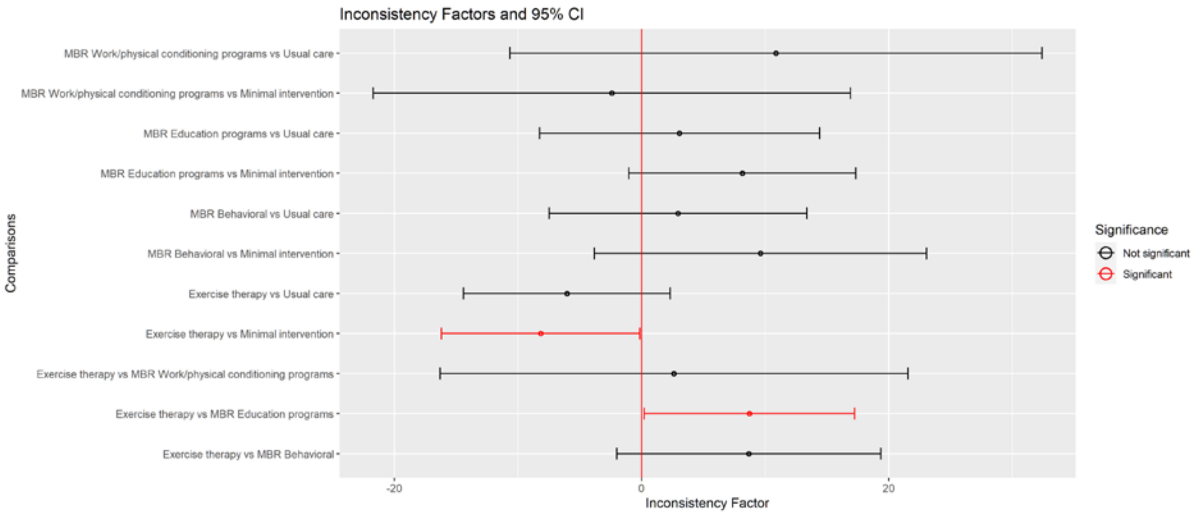

Frequentist Network meta-analysis of short-term disability outcomes

Table S6. Between, within and total homogeneity / consistency

	Q	Df	p-value
Total	316.89	70	<0.001
Within designs	282.52	65	<0.001
Between designs	34.37	5	<0.001

Table S7. Q statistic under the assumption of a full design-by-treatment interaction random effects model

	**Q**	**Df**	**p-value**	**τ**	**τ2**
Between designs	9.53	5	0.090	0.443	0.196

Figure S7. Local inconsistency assessment using inconsistency factors and 95% CI

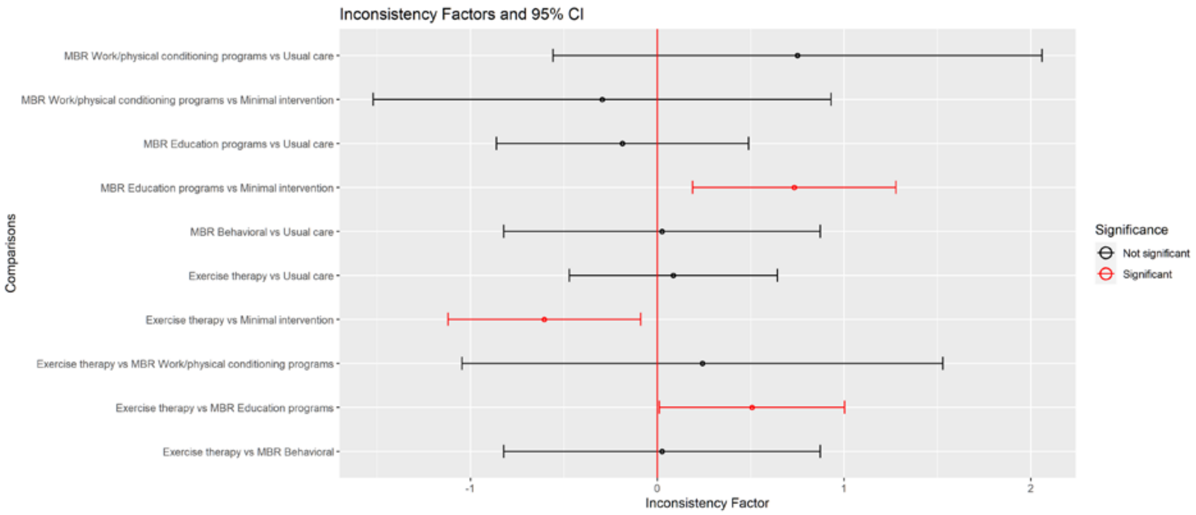

Figure S8. Funnel plot for NMA of pain outcomes

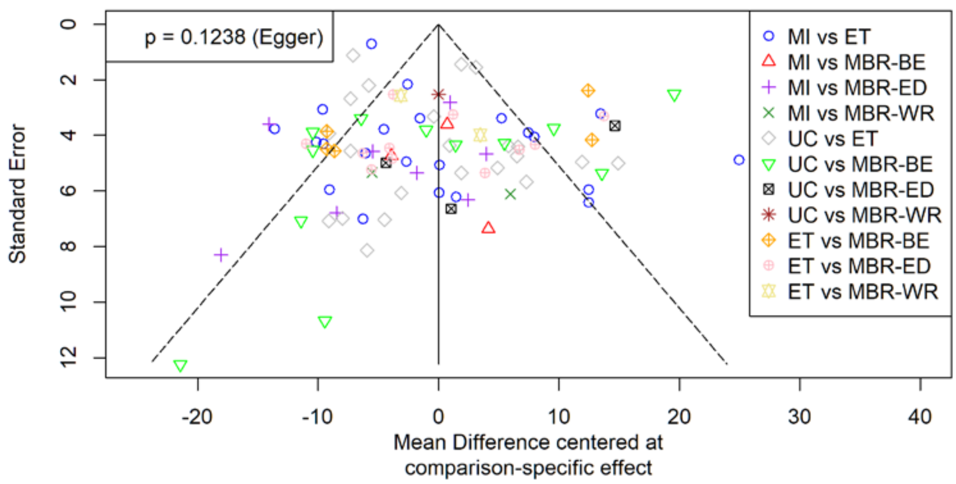

Figure S9. Funnel plot for NMA of disability outcomes

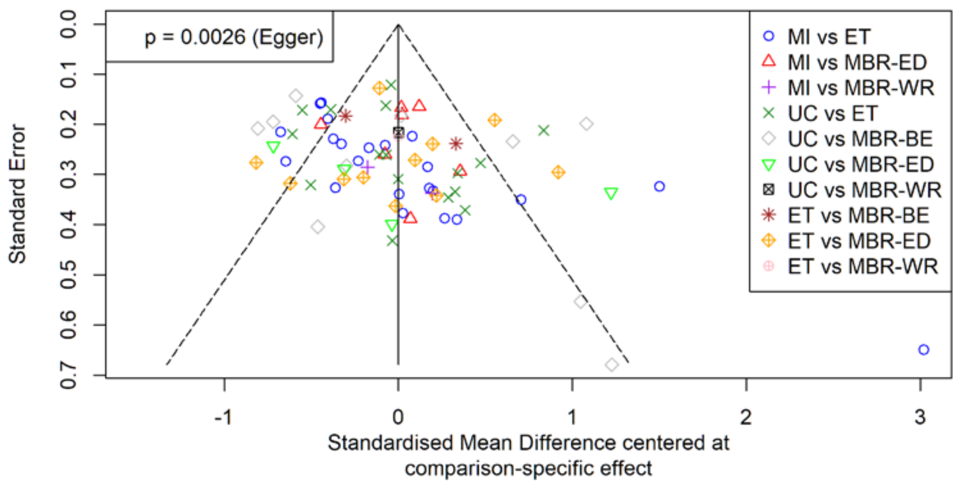

Bayesian Network Meta-Analysis of PAIN outcome

Figure S10. Node-splitting analysis of inconsistency for pain outcome

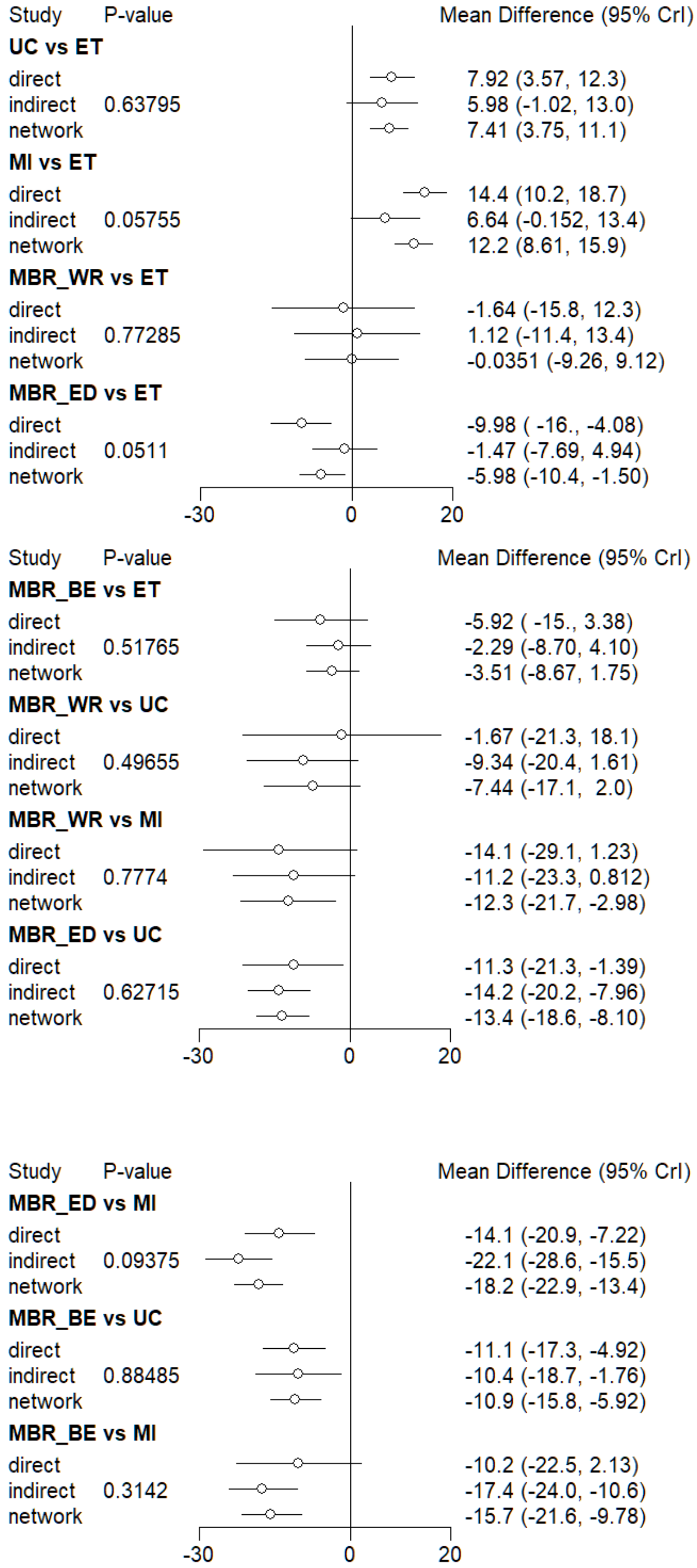

Figure S11. Probability rankings (pain outcome)

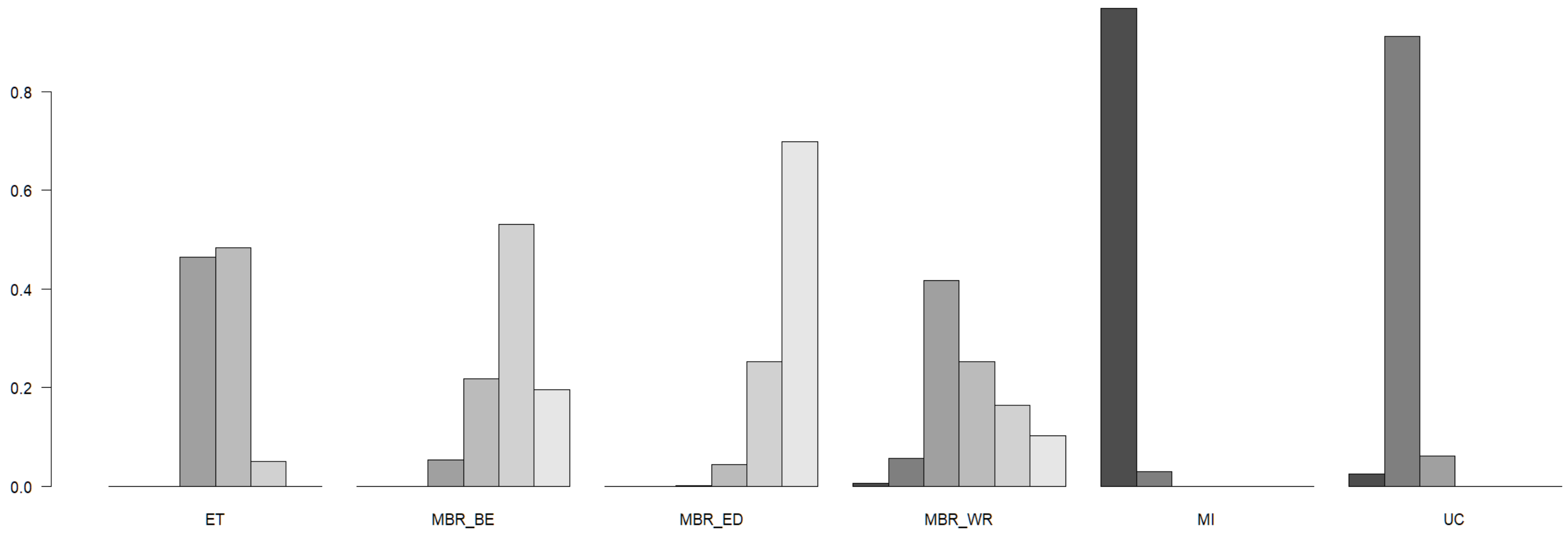

Figure S12. Forest plot of all treatment groups vs Minimal intervention (pain outcome)

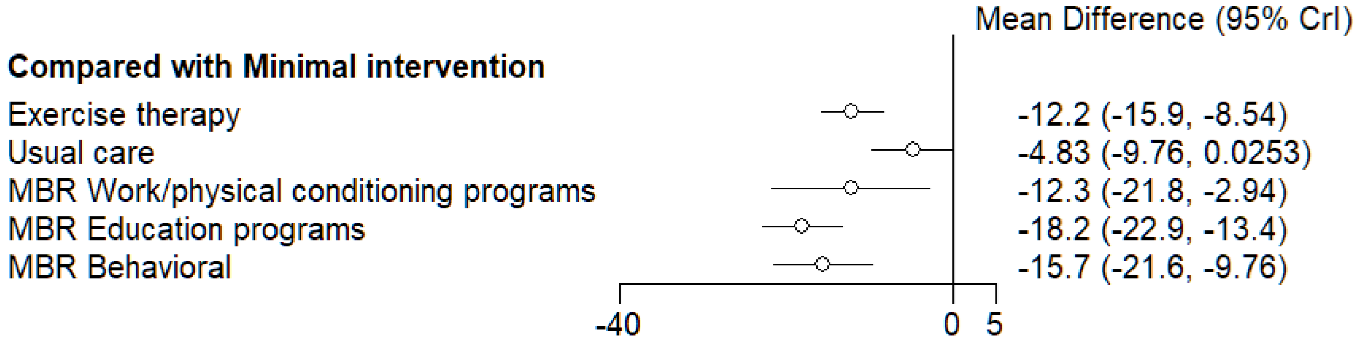

Bayesian Network Meta-Analysis of DISABILTY outcome

Figure S13. Node-splitting analysis of inconsistency for disability outcome

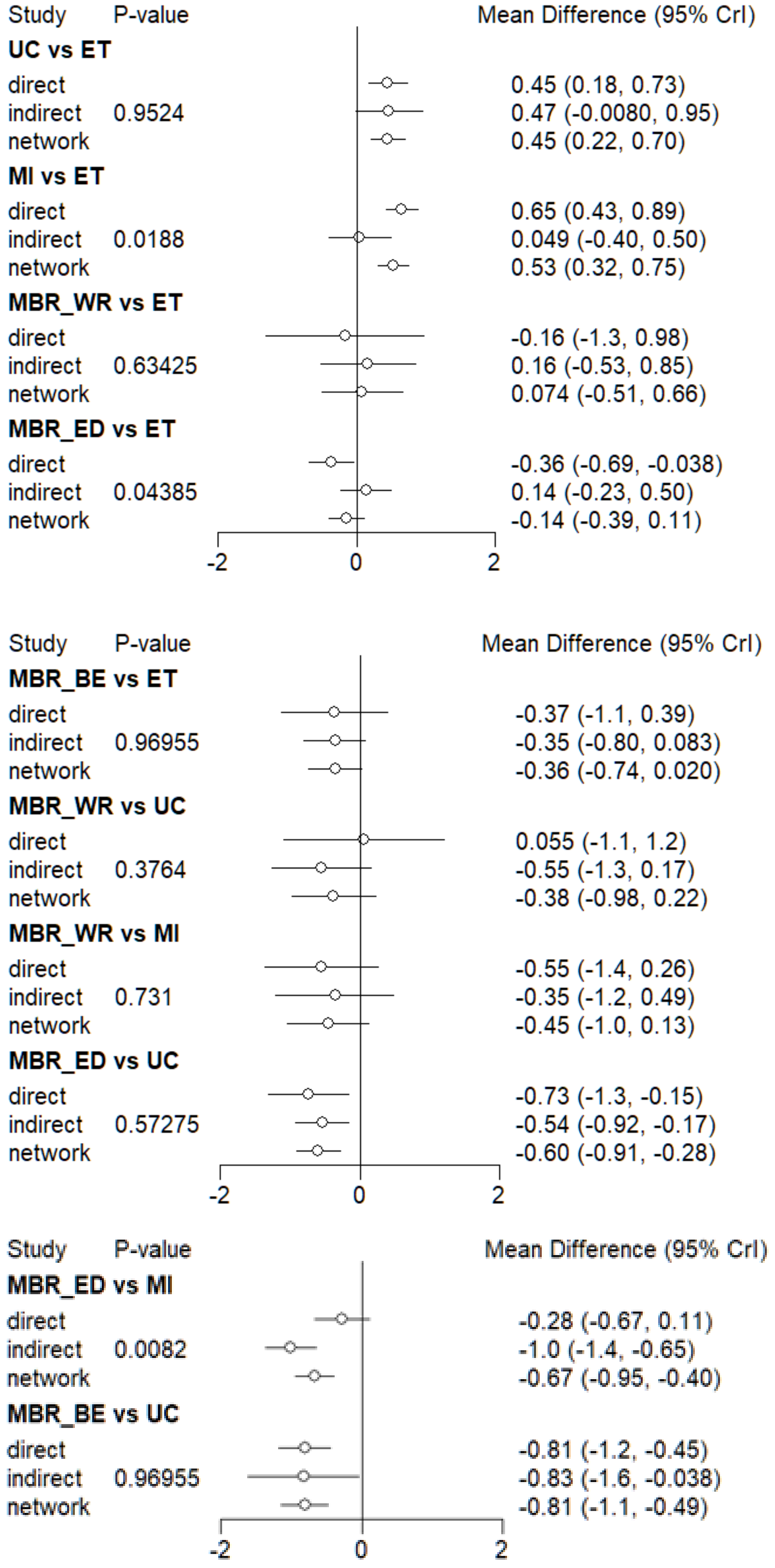

Figure S14. Probability rankings (disability outcome)

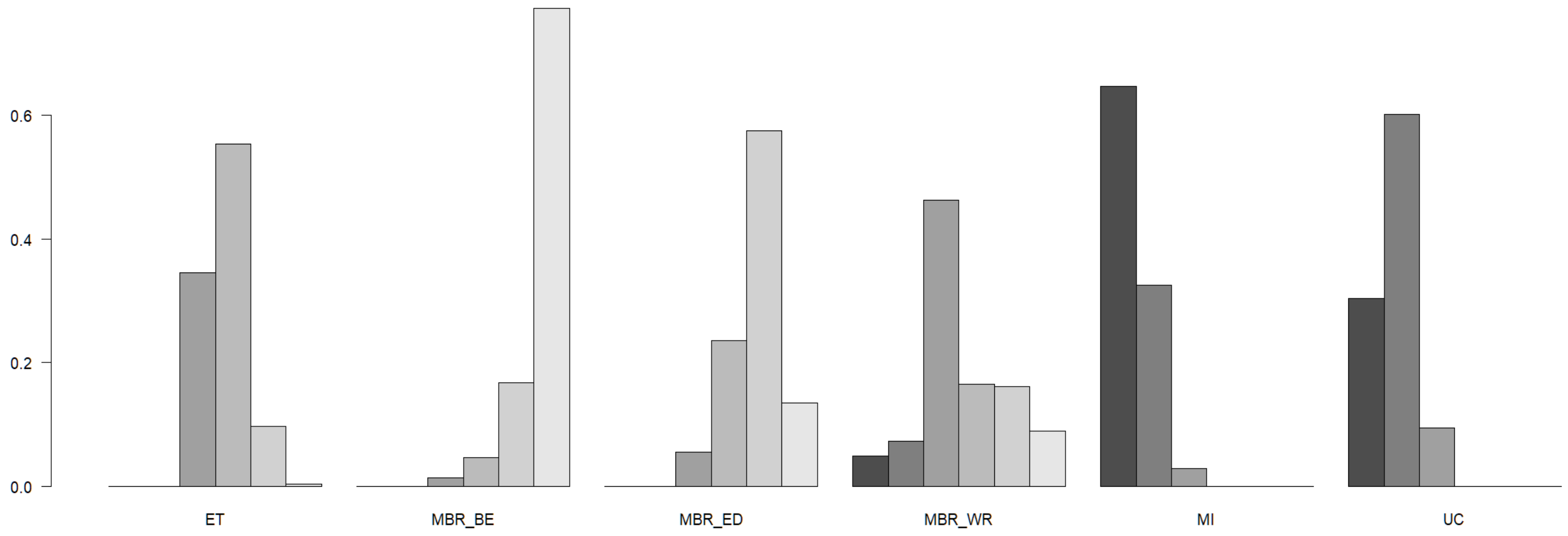

Figure S15. Forest plot of all treatment groups vs Minimal intervention (disability outcome)

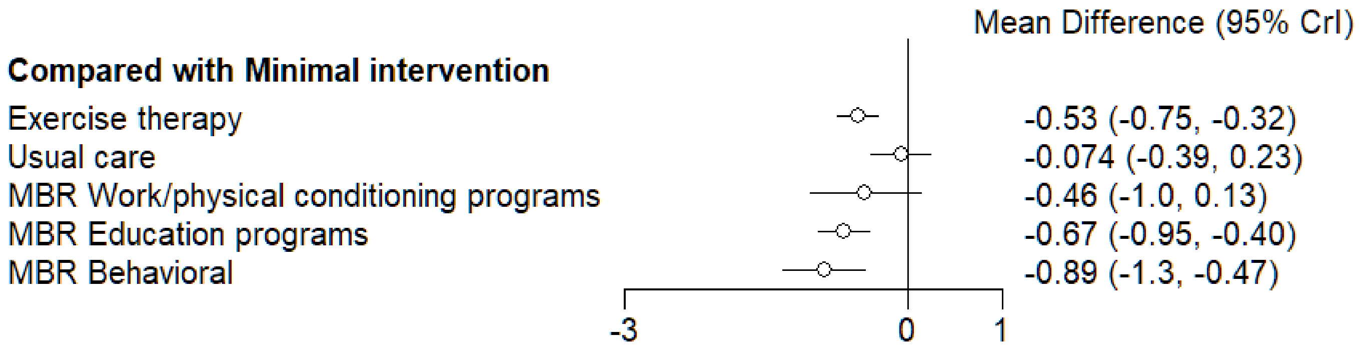

Supplement: Supplementary file 1 [file jcm-12-07489-s001.zip › jcm-2736126-supplementary.pdf]
